# A New Model for Raf Kinase Inhibitory Protein Induced Chemotherapeutic Resistance

**DOI:** 10.1371/journal.pone.0029532

**Published:** 2012-01-18

**Authors:** Fahd Al-Mulla, Milad S. Bitar, Jingwei Feng, Sungdae Park, Kam C. Yeung

**Affiliations:** 1 Department of Pathology, Health Sciences Center, Kuwait University, Faculty of Medicine, Safat, Kuwait; 2 Department of Pharmacology, Health Sciences Center, Kuwait University, Faculty of Medicine, Safat, Kuwait; 3 Department of Biochemistry and Cancer Biology, College of Medicine, University of Toledo, Toledo, Ohio, United States of America; University of Hong Kong, Hong Kong

## Abstract

Therapeutic resistance remains the most challenging aspect of treating cancer. Raf kinase inhibitory protein (RKIP) emerged as a molecule capable of sensitizing cancerous cells to radio- and chemotherapy. Moreover, this small evolutionary conserved molecule, endows significant resistance to cancer therapy when its expression is reduced or lost. RKIP has been shown to inhibit the Raf-MEK-ERK, NFκB, GRK and activate the GSK3β signaling pathways. Inhibition of Raf-MEK-ERK and NFκB remains the most prominent pathways implicated in the sensitization of cells to therapeutic drugs. Our purpose was to identify a possible link between RKIP-KEAP 1-NRF2 and drug resistance. To that end, RKIP-KEAP 1 association was tested in human colorectal cancer tissues using immunohistochemistry. RKIP miRNA silencing and its inducible overexpression were employed in HEK-293 immortalized cells, HT29 and HCT116 colon cancer cell lines to further investigate our aim. We show that RKIP enhanced Kelch-like ECH-associated protein1 (KEAP 1) stability in colorectal cancer tissues and HT29 CRC cell line. RKIP silencing in immortalized HEK-293 cells (termed HEK-499) correlated significantly with KEAP 1 protein degradation and subsequent NRF2 addiction in these cells. Moreover, RKIP depletion in HEK-499, compared to control cells, bestowed resistance to supra physiological levels of H_2_O_2_ and Cisplatin possibly by upregulating NF-E2-related nuclear factor 2 (NRF2) responsive genes. Similarly, we observed a direct correlation between the extent of apoptosis, after treatment with Adriamycin, and the expression levels of RKIP/KEAP 1 in HT29 but not in HCT116 CRC cells. Our data illuminate, for the first time, the NRF2-KEAP 1 pathway as a possible target for personalized therapeutic intervention in RKIP depleted cancers.

## Introduction

RKIP is a small evolutionary conserved protein that was first identified as a physiological inhibitor of the Raf-MEK-ERK pathway [1]. RKIP is well known for its metastasis suppression function in various cancer types. Loss or diminution of RKIP expression has been associated with increasing number of aggressive cancers [2], [3], [4], [5], [6], [7], [8]. Moreover, recent data implicated RKIP depletion in radio- and chemotherapeutic resistance both in *vitro* [9], [10], [11], [12], [13] and in *vivo* [14], [15]. The mechanisms behind such resistance remain elusive. Although current data implicate NF-κB activation by RKIP diminution as the most likely mechanism behind apoptotic cell death resistance. RKIP has been shown to negatively interfere with NF-κB signaling, by binding to NIK, TAK1, and TRAF6 [16], [17], [18]. Recently, elegant experiments illuminated the NF-κB-Snail-RKIP circuitry as possible mechanism for chemotherapeutic resistance in cancer cells [11] and that RKIP expression reversed cancer cells resistance to drugs and TRAIL induced apoptosis [10]. RKIP expression may be induced by chemotherapeutic drugs and this correlated with the onset of apoptosis [12]. Moreover, downregulation of RKIP by siRNA has been shown to confer resistance to anticancer drugs in inherently sensitive cancer cells [12].

Previously, we have reported that RKIP depletion in HEK-293 cells induced an intense reactive oxidative stress response that culminated in the activation of p38 and degradation of GSK3β protein [19]. It is well established that oxidative stress induces NRF2 activation through the uniquitination of KEAP 1 protein, which binds and inhibits NRF2 nuclear translocation [20], [21]. NRF2 is a transcription factor originally identified as a significant upregulator of intracellular antioxidants and phase II detoxification proteins containing ARE-cis acting element [22]. Later, growth, redox-regulating genes, ubiquitin-mediated proteosomal degradation and redox-related genes were identified as targets for NRF2 regulation [23], [24]. Therefore, NRF2 activation appears to confer cell survival benefit under detrimental environmental conditions. In cancer, NRF2 has been shown to be upregulated in more than 90% of head and neck squamous cancers [25] and was shown to determine chemoresistance in type II endometrial cancer [26]. Moreover, KEAP 1 was shown to be frequently inactivated by mutation or loss of heterozygosity in lung cancer [27], [28]. Given the intense induction of reactive oxygen species (ROS) in RKIP depleted HEK-499 cells, testing a hypothesis that links RKIP depletion or loss in cancers and immortalized cells with KEAP 1 destabilization and consequently NRF2 activation appears very attractive. Here, we report, for the first time, that KEAP1 protein expression in colorectal cancer is associated with RKIP stability and that the KEAP 1-NRF2 pathway may be a novel target in RKIP induced drug resistance.

## Results and Discussion

### RKIP-level modulation influences KEAP 1 protein in-*vitro*

KEAP 1 is a substrate adapter of a Cullin 3-based E3 ubiquitin ligase complex that binds NRF2 and degrades it in stress-free conditions through the proteosome pathway [29]. KEAP1 is the major sensor for ROS in cells. Upon exposure to oxidative stress, Cys 272 and Cys 288 get modified and Cys 226 and Cys 613 are induced to form intramolecular disulfide bond and another intermolecular disulfide bond at Cys 151 linking two KEAP 1 molecules together that culminates in the inactivation of KEAP1 and stabilization of NRF2 [30], [31], [32].

To elaborate on the relationship between RKIP and KEAP 1 proteins, we stably modulated RKIP level in *vitro*. The HEK-499 cells, derived directly from HEK-293 cells by miRNA silencing RKIP, and the Flp-In T-Rex-293 cells with Doxycycline inducible RKIP construct that elevate RKIP to slightly above physiological level [19], [33] were used in this study. We have chosen the HEK-293 immortalized cells to further investigate the RKIP-KEAP 1-NRF2-therapeutic resistance cascade because these cells have been well characterized (genetically and biochemically) by us.

**Figure 1:**
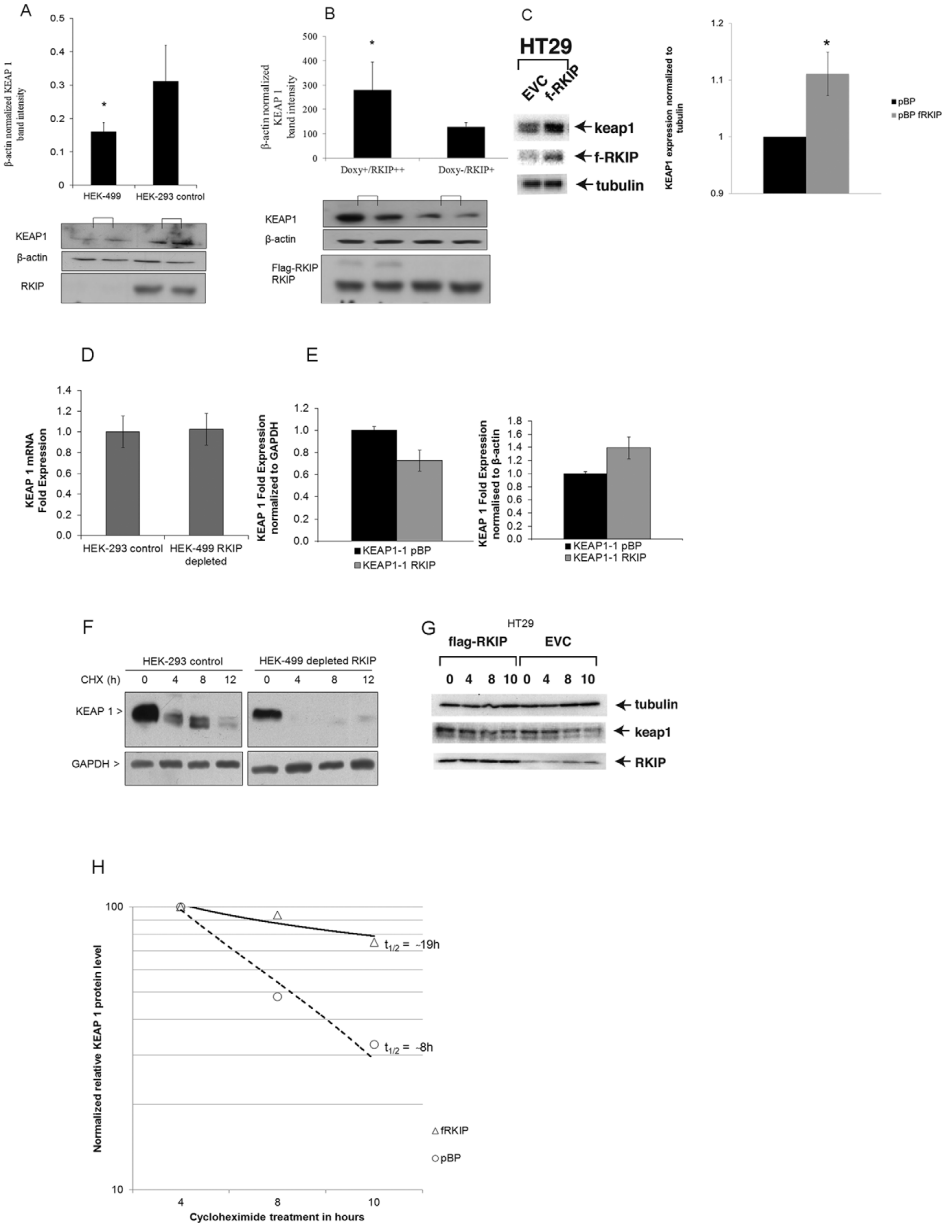
RKIP level modulation influences KEAP 1 protein expression and degradation. A, Western blotting (lower panel) and β-actin normalized densitometric measurements (upper panel) for KEAP 1 in HEK-499 and control cells. B, Western blotting and β-actin normalized densitometric measurement (bar charts) for KEAP 1 in RKIP induced cells (Doxy+/RKIP++ are Flp-In T-Rex 293 cells stimulated with Doxycycline and Doxy−/RKIP+ are not treated/stimulated). C, Western blot and densitometric measurements of KEAP 1 protein in HT29 CRC cells transfected with empty vector (EVC) or pBP vector that overexpresses flagged RKIP (fRKIP). D, Quantitative RT-PCR of normalized KEAP 1 mRNA fold changes in HEK-293 control and HEK-499 RKIP depleted cells. E, GAPDH (left) β-actin (right) normalized KEAP 1 fold changes in mRNA expression in HT29 control (KEAP1-1 pBP) and flagged RKIP (KEAP 1-1 RKIP) transfected cells. F, HEK-293 control and HEK-499 RKIP depleted cells exposed to cycloheximide (CHX; 35 µM) for the indicated times. KEAP 1 and GAPDH protein levels were analyzed by Western blotting. G, Western blotting for RKIP, KEAP 1 and tubulin in HT29 CRC cells transfected with empty vector (EVC) or pBP vector that overexpresses flagged RKIP (fRKIP) exposed to cycloheximide (CHX; 35 µM) for the indicated times. All western experiments were performed at least in duplicates and repeated twice. Asterisks indicate statistical significance (p<0.05 compared to equivalent controls).

In RKIP silenced HEK-499 cells, KEAP 1 protein was significantly underexpressed compared to HEK-293 control cells (Figure 1A). Conversely, inducing RKIP in Flp-In T-Rex-293 cells, stabilized KEAP 1 protein compared to uninduced cells (Figure 1B). Similarly, in HT29 colorectal cancer cells, the overexpression of RKIP stabilized KEAP 1 protein level (Figure 1C). However, this direct relationship between RKIP and KEAP 1 protein levels was not observed in the HCT116 CRC cell lines, that has codon-13 *Ki-Ras* oncogene mutation (data not shown), indicating that the proposed RKIP-KEAP 1 relationship may be a cell-specific phenomena. The basal-level changes in KEAP 1 protein were not due to altered transcription of the *KEAP 1* gene because silencing or induction of RKIP expression did not significantly affect KEAP 1 mRNA levels in HEK-499 or HT29 cells respectively (Figure 1D and 1E). These data indicate that RKIP modulation regulates KEAP 1 post-transcriptionally. To explore this further, we subjected HEK-293 and their RKIP-silenced derived cells (HEK-499) to protein synthesis inhibitor Cycloheximide for up to 12 hours. In control HEK-293 cells, the half-life of KEAP 1 protein was around 8 hours with significant degradation observed after 12 hours of treatment (Figure 1F). In RKIP depleted, HEK-499 cells, KEAP 1 half-life was less than 4 hours (Figure 1F). KEAP 1 protein degradation was even documented after 2 hours of Cycloheximide treatment (Supplementary Figure S1). These data strongly indicate that RKIP loss causes a rapid reduction in the basal level of KEAP 1 protein by accelerating its rate of degradation. Conversely, in HT29 cells, overexpressing RKIP (fRKIP), the half-life of KEAP 1 protein was significantly increased to around 19 hours compared to control HT29 cells (Figure 1, panels G and H).

### Antioxidant treatments rescue KEAP 1 protein expression in RKIP-silenced HEK-499 cells

**Figure 2:**
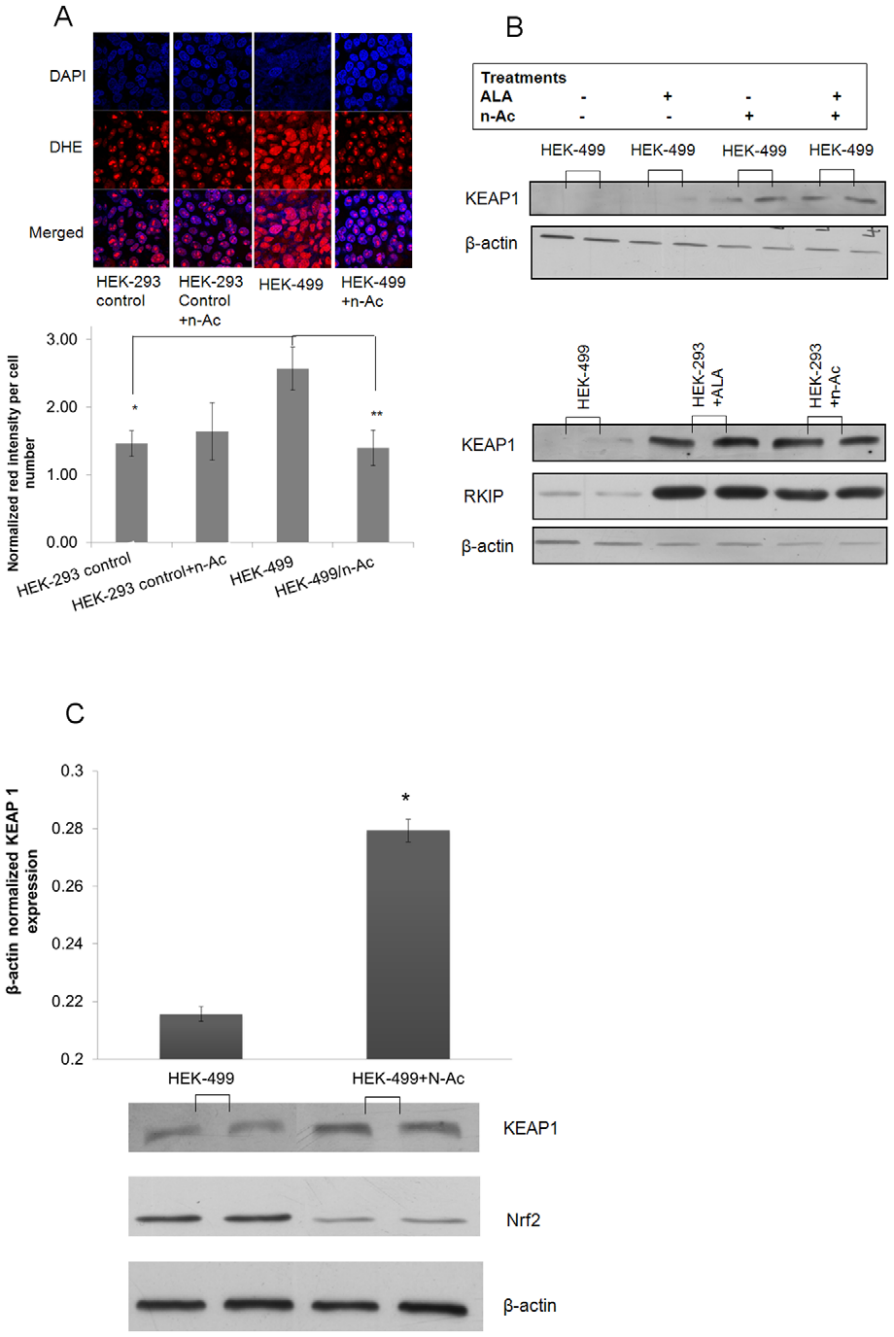
Antioxidant treatments rescue KEAP 1 in RKIP –depleted HEK-499 cells. A, Upper panels show confocal images of HEK-293 and 499 stained with dihydro-ethidium bromide (DHE) as an indicator of ROS before and after treatments with 5 mM N-Acetylcysteine (N-Ac). The lower bar chart represents the quantification of cell-number-normalized DHE intensities in depicted cells. B, Upper panel represents western blotting for KEAP 1 and β-actin loading control in HEK-499 cells before and after treatment of cells with the antioxidants N-Ac and/or ALA. The lower western panel depicts KEAP1 and RKIP protein levels in HEK-499 and HEK-293 after the indicated treatment. C, Densitometric measurements of β-actin normalized KEAP 1 expression in an additional independent experiment after treatment of cells with 5 mM of n-Ac for 16 hours, showing the rescue of KEAP 1 protein and concomitant reduction of total NRF2 in the treated HEK-499 cells. Western experiments were performed at least in duplicates and repeated twice. Asterisks indicate statistical significance (p<0.05 compared to equivalent controls).

The regulation of KEAP 1 protein is poorly understood. However, increased ROS are known to induce the ubiquitination of KEAP1 [34], [35]. We, therefore, examined ROS in HEK-499 and their equivalent control-HEK-293 cells using dihydro-ethidium bromide (DHE), a well-known, indicator of cellular ROS. Figure 2A shows a significant increase in ROS in HEK-499 cells when compared to corresponding control values. Consistent with these data is our previous findings, documenting that RKIP depletion induced H_2_O_2_ production in cells and the subsequent induction of antioxidant enzymes [19], [33], [36]. To establish whether RKIP reduces KEAP 1 by an oxidative stress mediated mechanism, we administered N-acetylcysteine (N-Ac) and/or α-Lipoic acid (ALA), two of the well-known antioxidants. [19]. As shown in Figure 2A, pretreatment of HEK-499 cells with N-Ac and/or ALA reduced the cellular levels of ROS as indicated by the decrease in DHE red color formation, More interestingly, these treatments in HEK-499 cells also significantly increased KEAP 1 protein content (Figure 2B and 2C) with subsequent reduction in NRF2 level, a transcription factor that is normally targeted for ubiquitination by KEAP 1 (Figure 2C). It is worthy of note that the above treatment regimen did not alter RKIP protein level in HEK-293 control cells (Figure 2B). Taken together, these data advance the notion that antioxidant treatment has the potential of rescuing KEAP 1 protein in RKIP depleted HEK-499 cells and that ROS may, partly, be the connecting nexus between RKIP and KEAP 1 in these cells. Although,, the increase in KEAP 1, albeit significant did not elevate KEAP 1 protein to levels seen in the original HEK-293 control cells, indicating that additional mechanism(s) may also be responsible for the RKIP protection of KEAP 1 from degradation. For example, RKIP may directly bind KEAP 1 or modulates other factors that protect KEAP 1from degradation. However, using protein-protein interaction microarray [19] and direct immunoprecipitation (Data not shown), we were unable to demonstrate any binding affinity between RKIP and KEAP 1 proteins. Recent data show that Sequestosome 1 (SQSTM1) is a binding partner of KEAP 1 and aids in its degradation [36]. Our microarray data obtained from silencing RKIP elucidated no relationship between RKIP modulation and SQSTM1 at mRNA level [33]. Therefore, more targeted experiments, using inducible RKIP promoter elements in a better suited cell lines, are required to confirm or offer alternative connections between RKIP-KEAP 1 proteins.

We next asked whether RKIP loss/reduction was also associated with decreased KEAP 1 level in a disease based Model in *vivo*. Accordingly, a colorectal cancer (CRC) was used as a model for two reasons: firstly, RKIP loss/reduction seems to occur in about 20% of this cancer [2] and secondly, KEAP1 expression has not been previously evaluated in colorectal cancer.

### Loss of RKIP expression correlates with diminution of KEAP 1 protein level in *vivo*

**Figure 3:**
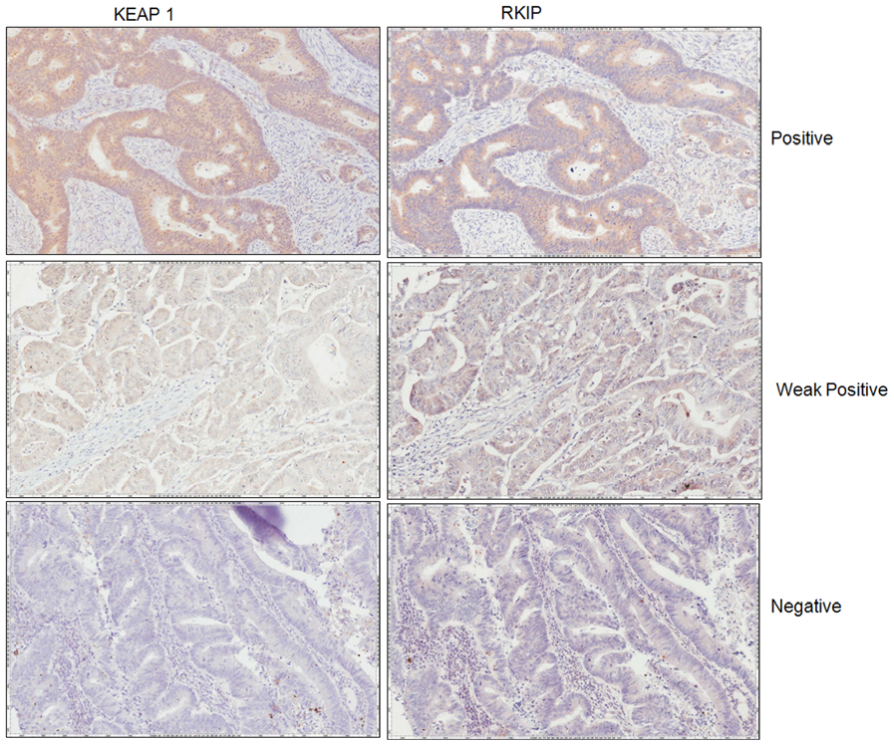
Immunohistochemical correlation between the expression of KEAP 1 and RKIP proteins in colorectal cancer tissues. Magnification is at 40×.

To verify our in *vitro* finding, and elaborate on the relationship between RKIP and KEAP 1 proteins, we studied the expression of these proteins in 105 CRC tissues using immunohistochemistry. We found that KEAP 1 expression was lost in 22 (21%) and diminished/reduced in 23 (21.9%) of the CRC cases (Figure 3). Moreover, our data elicited a significant association between the expression levels of RKIP and KEAP 1 in CRC tissues with Spearman's correlation of 0.42±0.09 S.E at p≤0.0001 (Table 1). KEAP 1 is frequently mutated in lung cancer and more so in adenocarcinomas [27]. Moreover, loss of heterozygosity at 19p13.2, where KEAP 1 is localized, occurred in 41% of non-small cell lung cancer cases [37]. Our data suggest that KEAP 1 protein loss or reduction is a significant event in colorectal cancer and that it may be causally related, in part, to RKIP expression loss or diminution in *vivo*. These data give credence to our in *vitro* findings. However, given the small size of this cohort, our data needs to be interpreted with caution and a larger cohort to further elaborate on the KEAP 1 protein/gene status in CRC, correlate its expression with clinicopathological parameters and its relationship to RKIP protein, is now warranted.

**Table 1:** Correlation between RKIP and KEAP 1 protein expression in 105 colorectal cancer tissues by immunohistochemistry.

RKIP n (%)			
	Negative 14(13)	Weak positive 34(32)	Positive 57(55)
KEAP 1 n (%)			
Negative 22 (21)	6 (5.7%)*	10 (9.5%)	6 (5.7%)
Weak Positive 23 (22)	5 (4.8%)	10 (9.5%)	8 (7.6%)
Positive 60 (57)	3 (2.8%)	14 (13%)	43 (41%)*

n = number of patients.

*Significant at p = 0.001 using two-sided Fisher's exact test.

### Loss of RKIP induces NRF2 nuclear accumulation

**Figure 4:**
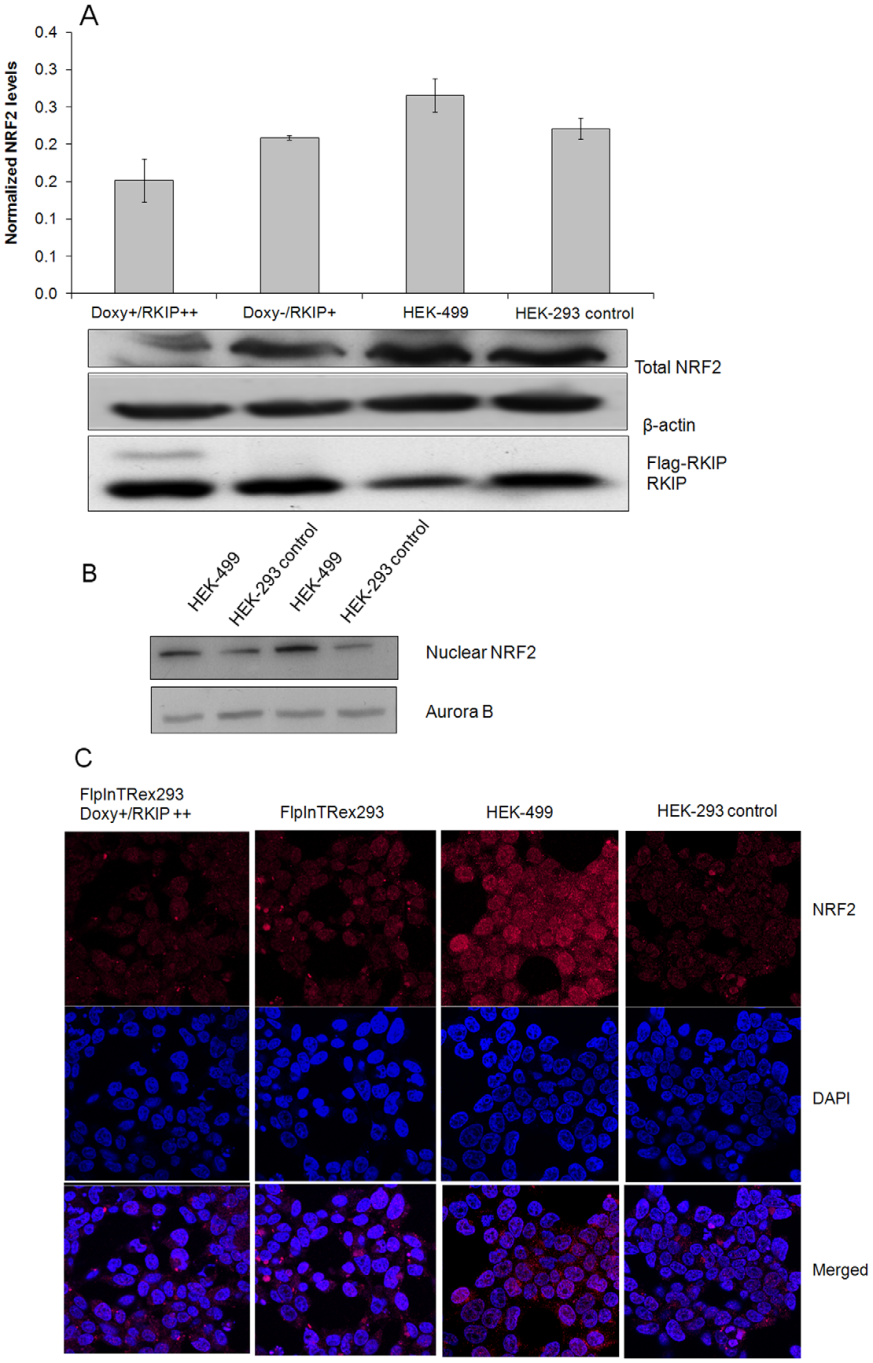
RKIP silencing induces nuclear translocation of NRF2. A, Shows western blotting and densitometric measurements (upper panel) for NRF2, RKIP and β-actin loading control from total protein extracts of depicted cells. B, Shows western blots on nuclear fractions of HEK-control and HEK-499 cells in duplicates with Aurora B as loading control. C, Immunofluorescent confocal microscopy for NRF2 in the depicted cells showing increased nuclear localization of NRF2 in RKIP depleted HEK-499 cells. Western experiments were performed in duplicates and repeated twice.

**Figure 5:**
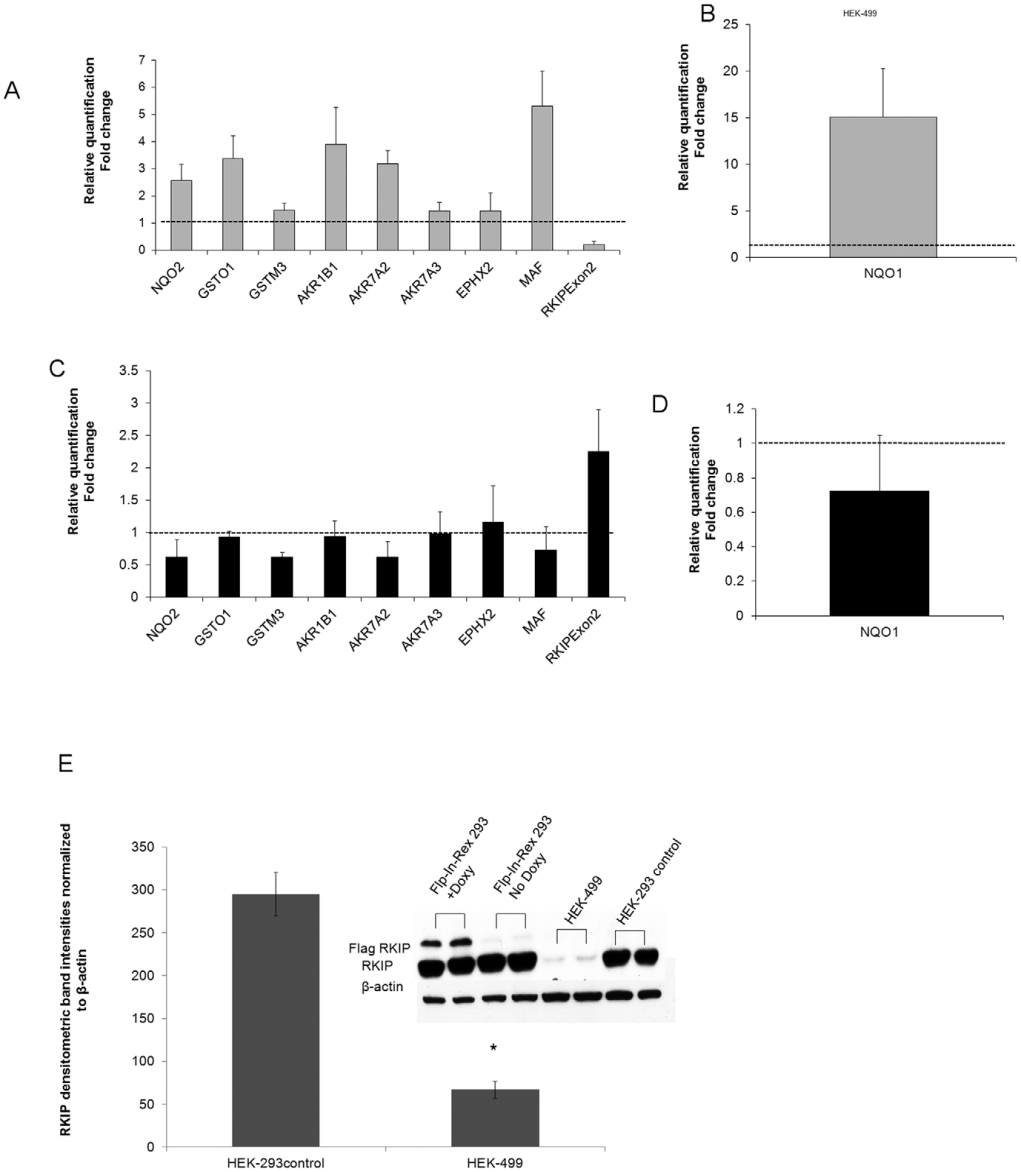
RKIP modulates NRF2-ARE containing genes. A, mRNA expression levels by RT-PCR of RKIP and 8 NRF2-inducible genes showing overexpression of genes involved in cellular protection from oxidative stress and chemotherapy in HEK-499 cells with silenced RKIP normalized to HEK-293 control cells. B, Shows fold changes in mRNA expression of the NQO1 gene in HEK-499 cells normalized to their equivalent control. C, mRNA expression levels of RKIP and the same 8 NRF2-inducible genes in Doxycycline induced Flip-in T-Rex 293 cells normalized to uninduced control cells. D, mRNA expression fold change of NQ1 gene in Doxycycline treated Flip-in T-Rex 293 cells normalized to untreated control cells. The gene names are shown on the X-axis. The fold change was calculated using ΔΔCT normalized threshold method. Error bars represent ±S.D. Dashed lines represent 1-fold change in expression. C, Repeat western blot and densitometric analysis of RKIP protein expression levels in the depicted cells confirming the RKIP-mRNA data shown in panel A. Asterisks indicate statistical significance (p<0.05 compared to equivalent controls).

ROS has been well established as a major activator of NRF2 through shifting the ubiquitination pathway towards KEAP 1 protein [21], [38]. We, therefore, examined NRF2 protein level in HEK-499 cells using western blotting. Total NRF2 was elevated in HEK-499 cells (Figure 4A), with concomitant increase of NRF2 in the nuclear fraction of HEK-499 cells compared to their controls (Figure 4B). This data was confirmed using confocal immunofluorescence (Figure 4C). Conversely, in RKIP overexpressing cells, NRF2 expression level was lower compared to uninduced cells (Figure 4A and 4C). NRF2 is a well characterized molecule that is known to enhance drug and therapeutic resistance through the transcriptional upregulation of antioxidants and phase II detoxification genes containing Antioxidant Response Elements (ARE-cis acting element) [22]. To further support our NRF2 data, we tested the RNA expression of NQO1 [39], GST family [40], AKR [41], MAF [42] and other molecules that have ARE *cis*-acting element and are known to be transcriptionally induced by NRF2 [22], [43]. Table 2 shows that genes related to phases I and II oxidation, reduction, hydrolysis and conjugation enzymes known to have chemopreventive properties, were upregulated at the mRNA level in HEK-499 cells (Figures 5A and 5B). Nine of these genes were also examined in doxycycline inducible cells overexpressing RKIP, normalized to uninduced cells and found to be either unchanged or underexpressed (Figure 5C and 5D). Quantitative RKIP mRNA and protein levels from the corresponding cells are shown in figure 5, panels A, C and E respectively.

**Table 2:** Genes involved in cellular protection found upregulated at mRNA level in HEK-499 RKIP depleted cells.

Cellular Function	Gene name (Gene symbol)	Expression fold-increase	P value*
Antioxidant proteins	Glutamate-cysteine ligase, modifier (GCLM) subunit	3.15	0.009
	Glutathione peroxidase 3 (GPX3)	3.03	0.028
	Glutathione reductase (GSR)	2.63	0.0026
	Glutathione synthetass (GSS)	3.35	0.0046
	NAD(P)H dehydrogenase, quinone 1 (NQO1)	2.7	0.01
	NAD(P)H dehydrogenase, quinone 2 (NQO2)	4.1	0.005
	Thioredoxin (TXN)	2.2	0.003
	Thioredoxin reductase 3 (TXNRD3)	2.5	0.0002
NADPH regeneration enzyme	Malic enzyme 1	6.3	0.001
Phase I drug oxidation, reduction and hydrolysis	Aldo-keto reductase (AKR) 1B	2.3	0.02
	Aldo-keto reductase (AKR) 7A3	2.25	0.001
	Aldo-keto reductase (AKR) 7A2	4.63	0.0003
	Carbonyl reductase 1 (CR1)	3.4	0.025
	Epoxide hydrolase 2 cytoplasmic (EPHX2)	2	0.001
Phase II drug conjugation	Glutathione S-transferase A4 (GSTA4)	2.8	0.0003
	Glutathione S-transferase (GSTP1) class pi	2.86	0.005
	Glutathione S-transferase omega 1	3.65	0.001
	Glutathione transferase zeta 1 (GSTZ1, maleylacetoacetate isomerase)	2.7	0.001
	Glutathione S-transferase M3 (GSTM3 brain^†^)	3.1	0.0006
	Microsomal glutathione S-transferase (MGST) 1	6.8	0.009
	Microsomal glutathione S-transferase (MGST) 2	2.24	0.002
	Microsomal glutathione S-transferase (MGST) 3	2.3	0.001
Transport	Multi-drug resistance-associated protein (Mrp4) ABCC4	3.8	0.01
	Multi-drug resistance-associated protein (Mrp6) ABCC6	4	0.02
	Solute carrier family 1(SLC1A5, Neutral amino acid transporter)	2.1	0.013
	Solute carrier family 2	2.2	0.03
	Solute carrier family 6	2.9	0.0003
	Solute carrier family 39	2.1	0.0003
Chaperones	Hsp40 (DnaJ)	3.4	0.00002
Growth factors	Fibroblast growth factor 13 (FGF 13)	4.44	0.0001
	peroxiredoxin family 1–5 (PRDX)	3.6	0.001
	Transforming growth factor β receptor (TGFBR3)	3.01	0.01
Transcription factors	v-maf musculoaponeurotic fibrosarcoma oncogene homolog avian (MAF)	3.4	0.03
	Peroxisome proliferator activated receptor delta (PPARD)	2.8	0.013

*ANOVA statistics was used to compare probe intensity means between test and control samples.

Our data, therefore, suggest that RKIP repression induces NRF2 nuclear accumulation and the transcriptional upregulation of NRF2-dependant drug resistant genes, giving further credence to our RKIP/KEAP 1-related findings. It is worthy of note that other, non KEAP 1-related mechanisms, can also influence NRF2 levels. For example, activated GSK3β is known to phosphorylate FYN, which accumulates in the nucleus and in turn phosphorylates NRF2 at Tyr-568 leading to its ubiquitination and degradation [44]. Our previous work also supports this Redox-divergent pathway as a plausible mechanism of NRF2 addiction since GSK3β was previously found inactivated in HEK-499 cells in response to RKIP silencing [19]. Our data illuminates NRF2 activation as a novel mechanism through which RKIP protein reduction or loss probably bestows resistance to therapy. We next tested this notion.

### Loss of RKIP augments chemoresistance in HEK-499 and HT29 cells

**Figure 6:**
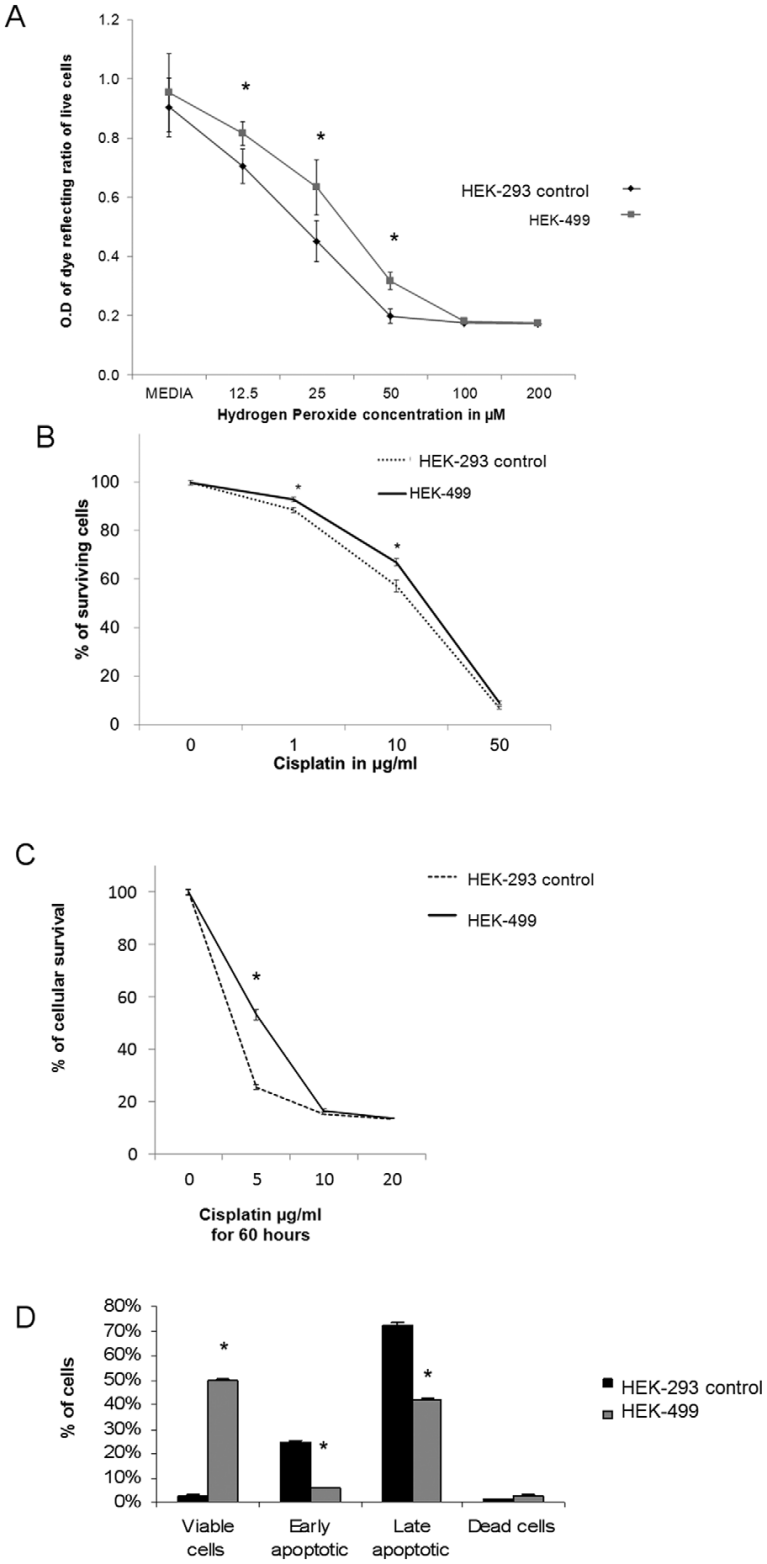
RKIP depletion induces hydrogen peroxide and chemotherapeutic resistance. A, WST assay showing enhanced survival of RKIP depleted HEK-499 compared to control cells with increasing hydrogen peroxide concentration. B, MTT assay/dose response curve depicting the chemosensitivity of HEK-293 and HEK-499 after treatment with various Cisplatin concentrations for 16 hours and (C) for 60 hours. D, Early apoptosis estimation using Anexin V of HEK-293 and 499 cells after treatment with 10 µg/ml Cisplatin for 16 hours demonstrating significant resistance of HEK-499 cells to apoptotic death. Asterisks indicate statistical significance (p<0.05 compared to equivalent controls).

To examine if RKIP silencing may bestow survival advantage to cells secondary to the overexpression of antioxidants and chemoprotective genes, we subjected HEK-499 cells to increasing concentrations of H_2_O_2_ or Cisplatin (Figure 6). Using an *in vitro* WST ELISA assay, we documented a significant survival advantage in HEK-499 cells (31.6% of surviving HEK-499 cells *versus* 19.7% in control cells) at H_2_O_2_ concentrations of 50 µM (Figure 6A). Conversely, the induction of RKIP by Doxycycline in Flp-In T-Rex-293 cells induced substantial cellular death (Supplementary Figure S2). This observation was not Doxycycline dependent since increasing Doxycycline concentration did not induce the expression of RKIP beyond 2.7 folds seen with 100 ng/ml of doxycycline treatment compared to uninduced cells. Data obtained from the Flp-In T-Rex-293 cells need to be interpreted cautiously because Doxycycline treatment may itself influence biochemical or other parameters that were not controlled for in this study (i.e the use of Doxycycline only on wild-type cells or cells without RKIP insert plasmids). Nevertheless, increasing Doxycycline concentration by 5-foldes did not influence apoptosis beyond what was expected from inducing RKIP by 2.7 folds (Supplementary Figure S2) Our data are in line with previous work showing that RKIP sensitizes cells to apoptosis [10], [12] and that, as anticipated, its loss or silencing bestows resistance to supra-physiological levels of H_2_O_2_. Similarly, using MTT assay, Figures 6B and 6C show that treating HEK-499 cells with increasing Cisplatin concentrations induced significantly less cytotoxicity in HEK-499 compared to control cells. We next utilized Annexin V to detect the effects of Cisplatin on the early apoptosis of these cells and showed that after 16 hours of 10 µg/ml Cisplatin treatment, about 50% of HEK-499 cells remained viable compared to 2.5% of control cells (Figure 6D).

**Figure 7:**
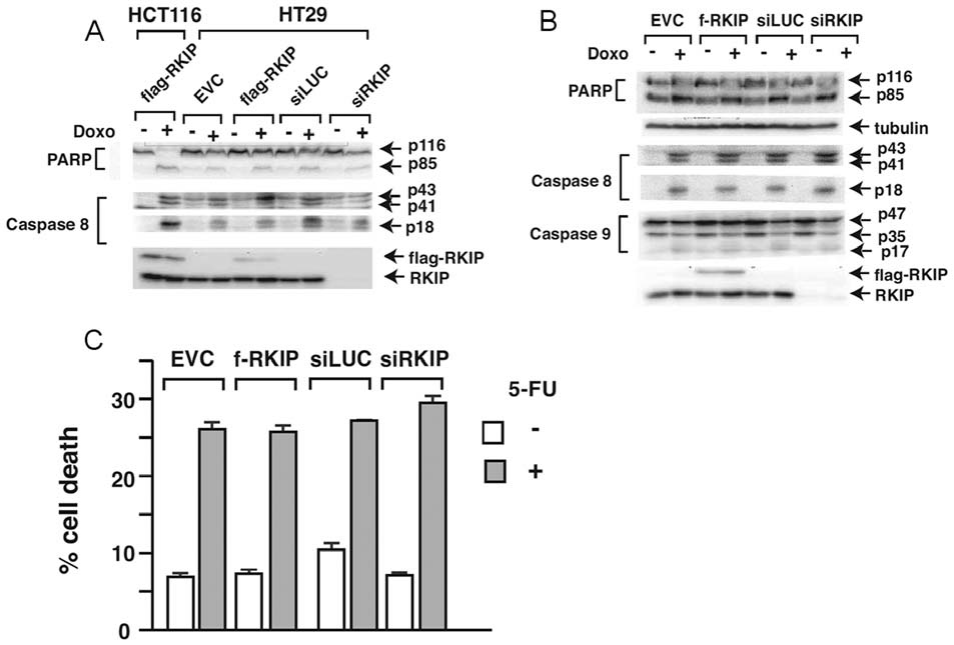
RKIP level modulations alters HT29 but not HCT116 CRC cells' sensitivity to chemotherapeutic agents. A, HT29 and B, HCT116 cells were stably transfected with different retroviral expression vectors as indicated. Transfected cells were treated with doxorubicin for 48 h before harvesting. Extracts were prepared for western immunoblot analysis with the specific indicated antibodies. C, HCTT116 cells were stably transfected with different retroviral expression vectors as indicated. Transfected cells were treated with 5-FU for 48 h. Extracts were prepared for cytometric analysis to examine binding to 7-ADD and annexin V.

We next examined the effect of RKIP expression on drug-induced apoptosis in colon cancer cell lines. We treated HT29 and HCT116 with the chemotherapeutic drug Doxorubicin (Adriamycin). Doxorubicin is a DNA-interacting drug that is widely used in the treatment of a wide range of cancers. Mechanistically, Doxorubicin inhibits topoisomerase-II, thereby introducing single- and double-strand breaks into the DNA molecule and induces apoptosis in cancer cells. The human colon carcinoma cell lines HT29 and HCT116 underwent extensive apoptosis when treated with doxorubicin (Fig. 7A). Apoptosis was measured by the cleavage of caspase-8, caspase-9 and PARP, a downstream physiological target of the caspase cleavage cascade (Fig. 7A). We also measured the drugs induced cell death by trypan blue staining and the results were consistent with the caspase and PARP cleavage studies (data not shown). Similar to the data obtained from the HEK-499 cell line, we observed a direct correlation between the extent of apoptosis and the expression levels of RKIP in HT29 cells (Figure 7A). In contrast, RKIP expression modulation had no effect on apoptosis as measured by caspases and PARP cleavage in HCT116 cells (Figure 7B). Similar results were observed when apoptosis were measured by trypan blue staining (not shown). To ensure the effect of RKIP is not drug specific, we also examined the effect of RKIP expression on 5-FU induced apoptosis in HCT116 cells. 5-FU is a drug commonly used in treatment of colon cancer. Unlike doxorubicin, 5-FU induces apoptosis in cancer cells by a different mechanism. 5-FU is a pyrimidine analog and functions as an inhibitor of thymidylate synthase, which is an important enzyme in DNA replication. As previously reported treatment of HCT116 with 5-FU for 48 h induced extensive apoptosis (cell death) as measured by binding to 7 Amino-Actinomycin D (7-AAD) and annexin V (Figure 7C). Similar to what was observed with doxorubicin, expression of RKIP had no effect on apoptosis induced by 5-FU. Our Data indicate that the effect of RKIP on apoptosis and chemoresistance is cell specific in that cells like HCT116, which harbor activated Ki-Ras, may not be influenced by molecules, like RKIP, that inhibit the MAPK-pathway. Indeed, resistance to Cetuximab, an EGFR blocker, has been well-documented and contraindicated in Ki-Ras driven CRC [45].

**Figure 8:**
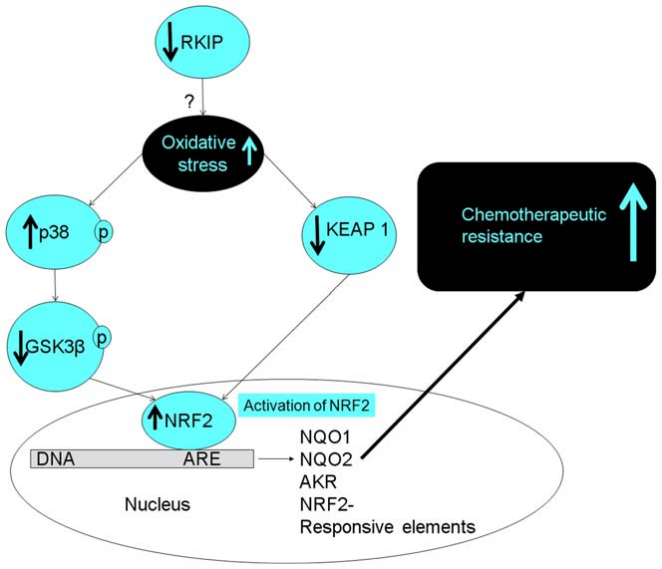
A proposed model for RKIP-induced drug resistance. In unstressed normal cells and in the presence of abundant RKIP, NRF2 is kept in the cytoplasm by KEAP 1 binding and shuttled outside of the nucleus by GSK3β, phosphorylated FYN protein. Upon loss or the reduction of RKIP in a cell [19], inactivation/ubiquitination of KEAP1 ensues. In addition, RKIP loss/depletion causes the inactivation of GSK3β through phosphorylation at its T390 residue by p38 [19]. Both events, ? which may be partly oxidative stress driven, culminate in the stabilization of NRF2 [30], [31], [32]. This consequently, enhances the transcription of antioxidants and phase II detoxification genes containing ARE-cis acting element culminating in the induction of drug resistance.

A proposed model summarizing the consequences of RKIP loss or reduction and its association with therapeutic resistance is shown in Figure 8.

Overall, our data have important ramifications for the treatment of cancers characterized by RKIP reduction or loss in that pharmacological or other interventions, which augment the KEAP 1 related pathways or directly target NRF2 may be of potential value in future personalized therapy.

## Materials and Methods

### Ethics Statement

The project number 2006-1302-07 has been ethically reviewed by the Ethical committee of the Faculty of Medicine and approved on 13^th^ April 2008. The immunohistochemistry procedure used on human colorectal cancer tissues was in accordance with the ethical standards implemented at University of Kuwait. We have not used any de novo cell lines. All cell lines used were described previously or referenced. No human subjects or vertebrates were used in this study.

### Cell culture and treatments

The generation of RKIP silenced and inducible cells, plasmid designs, transfection, and selection protocols have been described previously [33]. Flp-In T-Rex-293 cells were originally purchased from Invitrogen (Paisley, UK) and authenticated by them (http://tools.invitrogen.com/Content/SFS/ProductNotes/F_051025_Flp-In-TS-TL-MKT-HL.pdf). HEK-293 cell lines were originally purchased from CRUK and were authenticated by the European Collection of Cell Cultures (ECACC) in September 2009 using microsatellite genotyping (PCR-based). The parental cell line for the HEK-499 derived cells is the aforementioned HEK-293 cell line. All cells were used for experiments for 6–8 weeks before they were replaced with fresh stocks, which are stored in liquid nitrogen. HEK 293 (Human embryonic kidney cells), were grown in 1× DMEM (Gibco, Invitrogen, Grand Island, NY, U.S.A) supplemented with 10% FBS (Gibco, Invitrogen, Grand Island, NY, U.S.A). The HEK-499 cells containing the miRNA vector pcDNA™6.2-GW/EmGFPmiR-PBP_miR_499 were cultured in 1×DMEM media supplemented with 5 µg/ml Blasticidin S HCL (Sigma, Carlsbad, CA, USA) and incubated at 37°C, with 5% CO_2_. The Flp-In T-Rex-293 cell line (Gibco, Invitrogen, Grand Island, NY, U.S.A) transfected with 1 µg pcDNA5/FRT-FLAG-RKIP and 9 µg pOG44 (Invitrogen, V6005-20) were cultured in 1× DMEM (GIBCO®, Invitrogen Grand Island, NY, USA), supplemented with 10% Tet-free approved FBS (Clontech, Mountain View, CA, U.S.A). Switching on RKIP expression was achieved with 100 ng/ml doxycycline (Sigma, Carlsbad, CA, USA) for 48 hours prior to initiating the experiments.

The human colorectal cell lines HT29 was obtained from the ATCC and cultured in McCoy's 5A media supplemented with 10% FBS. The RKIP cDNA or empty vector control was introduced into HT29 by retrovirus transduction as previously described [12]. To determine the half-life of KEAP 1 protein, HEK-293, HEK-499 and HT29 cells were treated with 35 µM Cycloheximide, a *de novo* protein synthesis inhibitor for 10–12 hours.

### Immunohistochemistry

Sections from formalin-fixed paraffin-embedded colon cancer were cut into 4 µm thick sections, deparaffinized in three changes of xylene, and rehydrated through descending graded alcohols to water. Antigen retrieval was achieved by automated pressure cooker in 10 mmol/L of sodium citrate buffer at pH 6. Slides were then rinsed in running water for 5 minutes. Endogenous peroxidase was blocked with 3% hydrogen peroxide in water for 15 minutes at room temperature. The slides were then rinsed in water and immersed in PBS for 15 minutes at room temperature followed by blocking in PBS with 5% goat serum (Vector Laboratories, Inc., CA, U.S.A) for 1 hour. Sections were then incubated with rabbit polyclonal antibody against RKIP protein (dilution 1∶900), or rabbit monoclonal antibody against KEAP 1 (dilution 1∶100; Cell Signaling, Danvers, MA) diluted with Antibody Diluent (S 0809; DAKOCytomation Norden A/S, Glostrup, Denmark) and incubated overnight in a humidified chamber at 4°C. After washing 3-times with PBS, the slides were incubated with biotinylated secondary antibody at a dilution of 1∶500 for 30 minutes (Biotinylated Anti-Rabbit IgG, Vector Laboratories, Inc., CA, U.S.A). To detect the stain, VECTASTAIN Elite ABC system (Vector Laboratories, Inc., CA, U.S.A) was used following the manufacturer's instructions. Slides were imaged and quantified using an area and intensity scores described previously [46].

### Western Blotting

Cells at 70% confluency were lysed for 5 minutes on ice in lysis buffer (20 mM Tris-HCl pH 7.5, 150 mM NaCl, 1 mM Na_2_EDTA, 1 mM EGTA, 1%Triton-X100, 2.5 mM sodium pyrophosphate, 1 mM β-glycerophosphate, 1 mM Na_3_V0_4_, and 1 µg/ml leupeptin). Total cell lysates or nuclear fractions (prepared by using NE-PER extraction kit from Thermo Scientific, (Rockford IL, U.S.A) using the manufacturer's protocol) were sonicated briefly and centrifuged for 10 minutes at 14,000×g at 4°C. An aliquot was removed to determine the protein concentration (Thermo Scientific, Rockford, IL, U.S.A, BCA protein Assay kit Reducing Agent Compatible). Equal amount of protein extracts were separated by sodium dodecylsulphate– polyacrylamide gel electrophoresis (SDS-PAGE) and transferred to polyvinylidene fluoride (PVDF) membranes in transfer buffer (25 mM Tris-HCl, 150 mM glycine, 20% v/v methanol and 0.1% w/v SDS, pH 8.3) at 100 V at 4°C. Membranes were blocked for 1 hour at room temperature with 10% non-fat dry milk in Tris buffered saline-Tween (TBST) solution (20 mM TrisHCl pH 7.6, 137 mM NaCl, 0.1% v/v Tween-20). Membranes were incubated overnight at 4°C with 10 ml of rabbit polyclonal KEAP 1 or NRF2 or RKIP antibodies (Santa Cruz Biotechnology, CA, U.S.A) diluted in TBST with 5% w/v non-fat dry milk. After 3 washes with TBST membranes were incubated with horse radish peroxidase coupled goat anti-rabbit secondary antibody diluted 1∶10,000 in TBST for 2 hours at room temperature, and washed with TBST. Detection was by chemiluminescence using a commercial kit (Super Signal West Pico chemiluminescent Substrate, Pierce). Membranes were stripped and rehybridized with β-actin (Imigenex, CA, U.S.A) or Aurora B for nuclear extracts (BD transduction, MD, U.S.A) to account for equal loading. Developed films were scanned and quantified using the Quantity one software of the GS-800 calibrated densitometer (Bio-Rad, CA, U.S.A).

### Confocal microscopy

5×10^4^ of Cells were grown at 37°C in a humidified CO_2_ incubator until 70% confluency was reached. 1–2 ml of cell suspension were washed twice in phosphate PBS at room temperature, then fixed with 4% paraformaldehyde in PBS (pH 7.4) for 15 minutes at room temperature. After 3 washes with PBS, cells were permeabilized by incubation in PBS containing 0.1% Triton X-100 for 15 minutes at room temperature, followed by 3 washes in PBS. Cells were then blocked by incubation in PBS containing 1% bovine serum albumin (BSA) (Sigma, St. Louis, MO, U.S.A) for 1 hour at room temperature. The primary rabbit polyclonal antibody for NRF2 (Santa Cruz, CA, U.S.A) was diluted 1∶100 in PBS with 1% BSA (Sigma, St. Louis, MO, U.S.A) and incubated at 4°C overnight. After 3 washes in PBS for 5 minutes each, anti-rabbit specific fluorophore-conjugated secondary antibodies (1∶500 dilution) was added for 1 hour at room temperature. Coverslips were then washed 3 times in PBS for 5 minutes each, and visualized by LSM 510 META confocal microscope (Carl Zeiss, Germany).

### RT-PCR

Taqman real-time PCR Assay on Demand pre-designed plates with accession numbers given in Figure 1 (Life Technologies, Carlsbad, CA, U.S.A) were used to study NRF2 responsive genes. Each transcript was assayed in triplicate and normalized to, GAPDH (HS-99999905_ml) or 18S (HS-99999901_S1) housekeeping genes using the equivalent control cells as calibrators. cDNA synthesis was performed using High-Capacity cDNA Reverse Transcription Kit (Life Technologies, Carlsbad, CA, U.S.A) following the manufacturer's protocol. The reverse transcription conditions were: Step1–25°C for 5 minutes, step2–37°C for 120 minutes and step3–85°C for 5 minutes. PCR assays were performed using Taqman Gene Expression Master Mix (Life Technologies, Carlsbad, CA, U.S.A) on 7500 ABI using the following conditions; 1 cycle of 95°C for 10 minutes, followed by 40 cycles at 95°C for 15 seconds then 60°C for 1 minute. For relative quantification, the comparative CT method, given by the formula 2^−ΔΔCT^, was utilized with the amount of target, normalized to at least two separate endogenous references and relative to a calibrator [33].

### Cellular proliferation and death assays

The effects of Cisplatin on cellular proliferation were determined by the 3-(4,5-dimethylthiazol-2-yl)-2,5-diphenyltetrazolium bromide (MTT) uptake method. For H_2_O_2_ effects on cells, H_2_O_2_ ranging from 0–200 µM was added to cells growing in complete Media. Cell proliferation assay was performed using the spectrophotometric cell proliferation reagent WST-1 following the manufacturer's protocol (Roche, IN, U.S.A). Early indicator of apoptosis after 16 hours treatment of Cisplatin at 10 µg/ml was detected by flowcytometry using the Annexin V- FITC/7AAD kit (Beckman Coulter, CA, U.S.A) following the manufacturer's instructions. The cell preparations were analysed immediately aided by the FC 500 flowcytometer (Beckman Coulter, CA, U.S.A).

### Statistics

SPSS software version 17 was used for statistical calculations. Data are expressed as mean ± S.D. Contingency table statistical associations were calculated using two-sided Chi-square or Fisher's exact tests. Two-sampled Student's *t*-test assuming unequal variances was used to compare mean-data between two groups. ANOVA statistics was used to compare means of 3 groups or more. A level of p≤0.05 was considered to be significant.

## Supporting Information

Figure S1 Western blotting for KEAP 1 and GAPDH in HEK-499 (left) and HEK-293 (right) from cells exposed to cycloheximide (CHX; 35 µM) for the indicated times.
(TIF)

Figure S2 WST assay showing reduced survival after RKIP induction by 100 ng/ml doxycycline treatment in Flp-In T-Rex-293 cells. Increasing Doxycycline concentration did not influence RKIP induction or cellular death. Asterisks indicate statistical significance (p<0.05 compared to untreated cells).
(TIF)
