# Supplementary figures and images for: A New Model for Raf Kinase Inhibitory Protein Induced Chemotherapeutic Resistance
Source: PLoS One. 2012 Jan 18;7(1):e29532. doi: 10.1371/journal.pone.0029532 (PMC3261143; doi:10.1371/journal.pone.0029532)

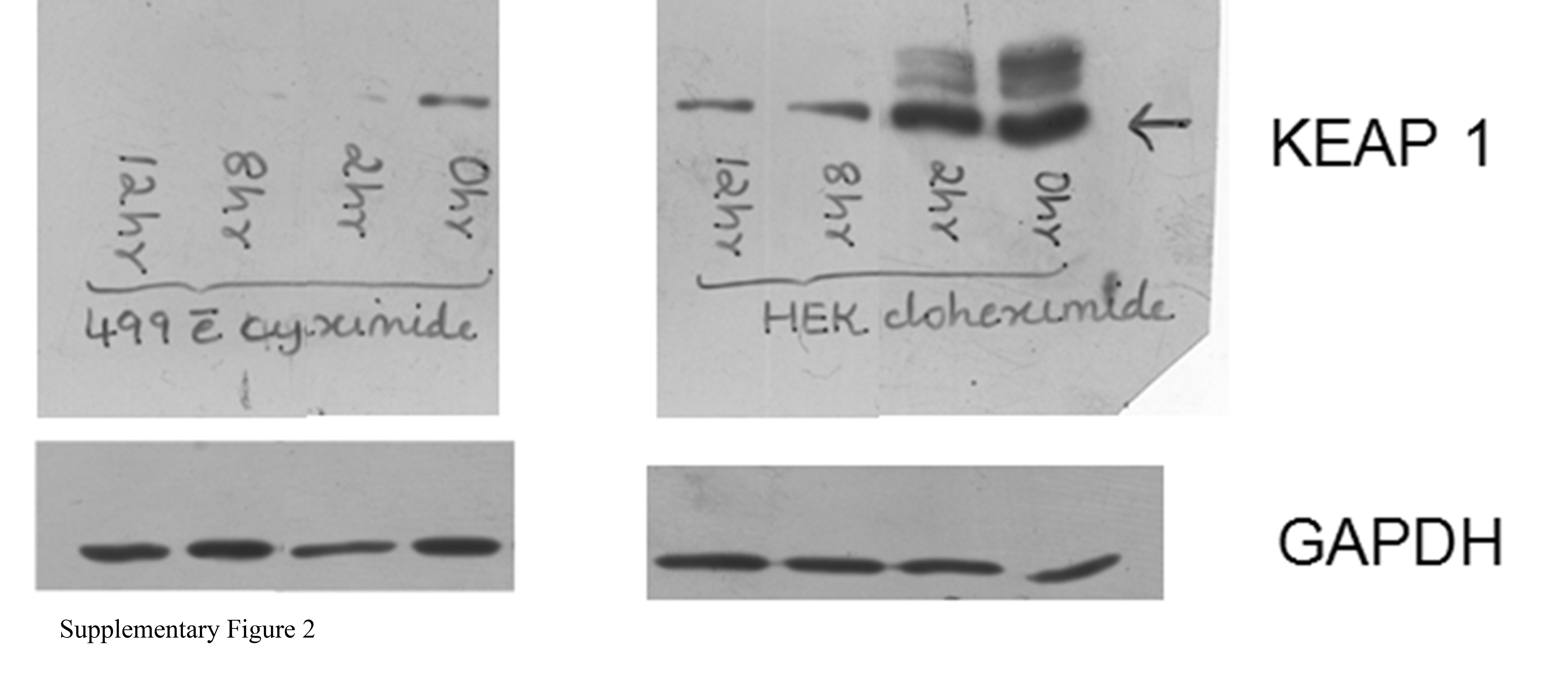

Supplement: Figure S1 — Western blotting for KEAP 1 and GAPDH in HEK-499 (left) and HEK-293 (right) from cells exposed to cycloheximide (CHX; 35 µM) for the indicated times. (TIF) [file pone.0029532.s001.tif]

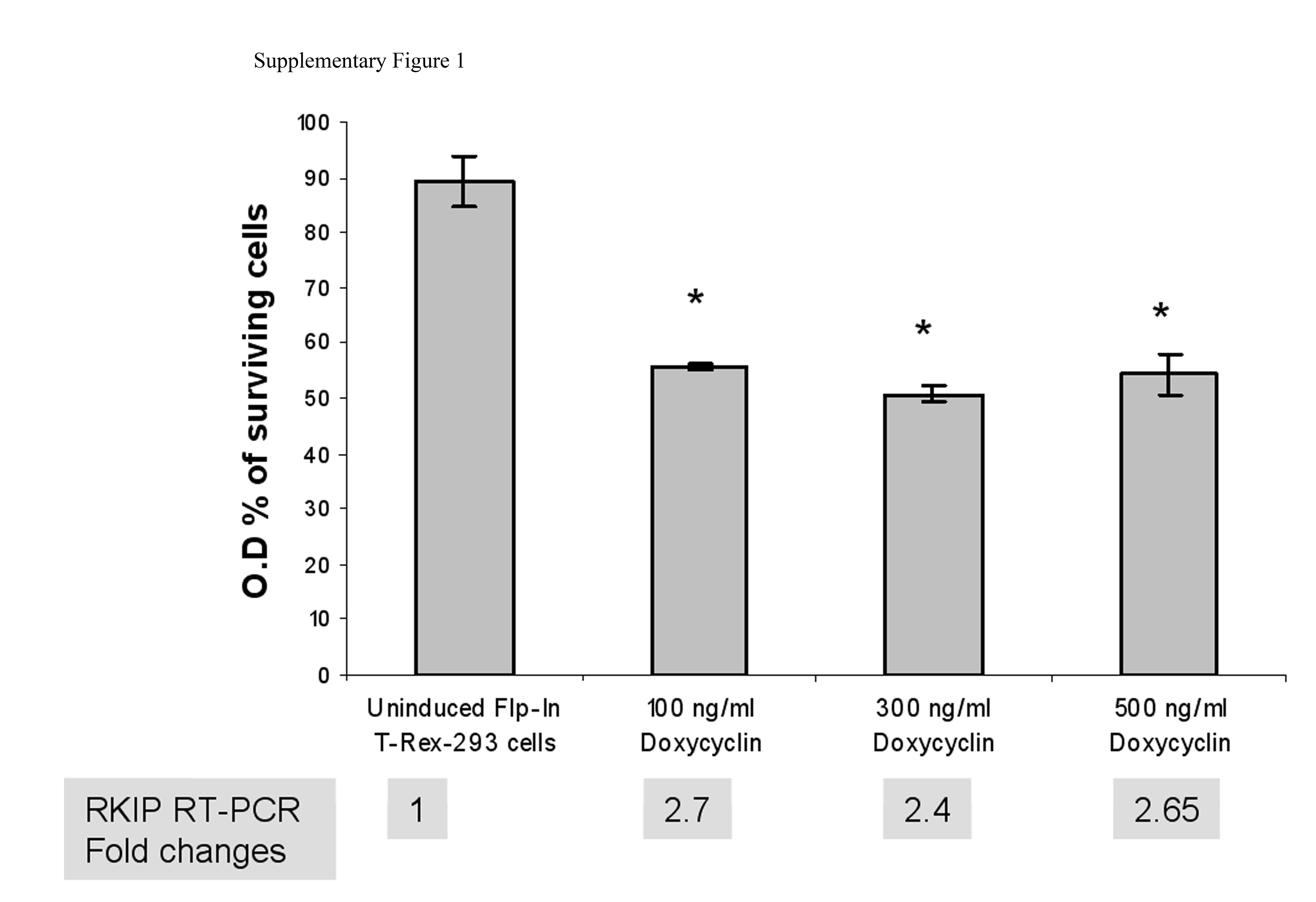

Supplement: Figure S2 — WST assay showing reduced survival after RKIP induction by 100 ng/ml doxycycline treatment in Flp-In T-Rex-293 cells. Increasing Doxycycline concentration did not influence RKIP induction or cellular death. Asterisks indicate statistical significance (p<0.05 compared to untreated cells). (TIF) [file pone.0029532.s002.tif]
